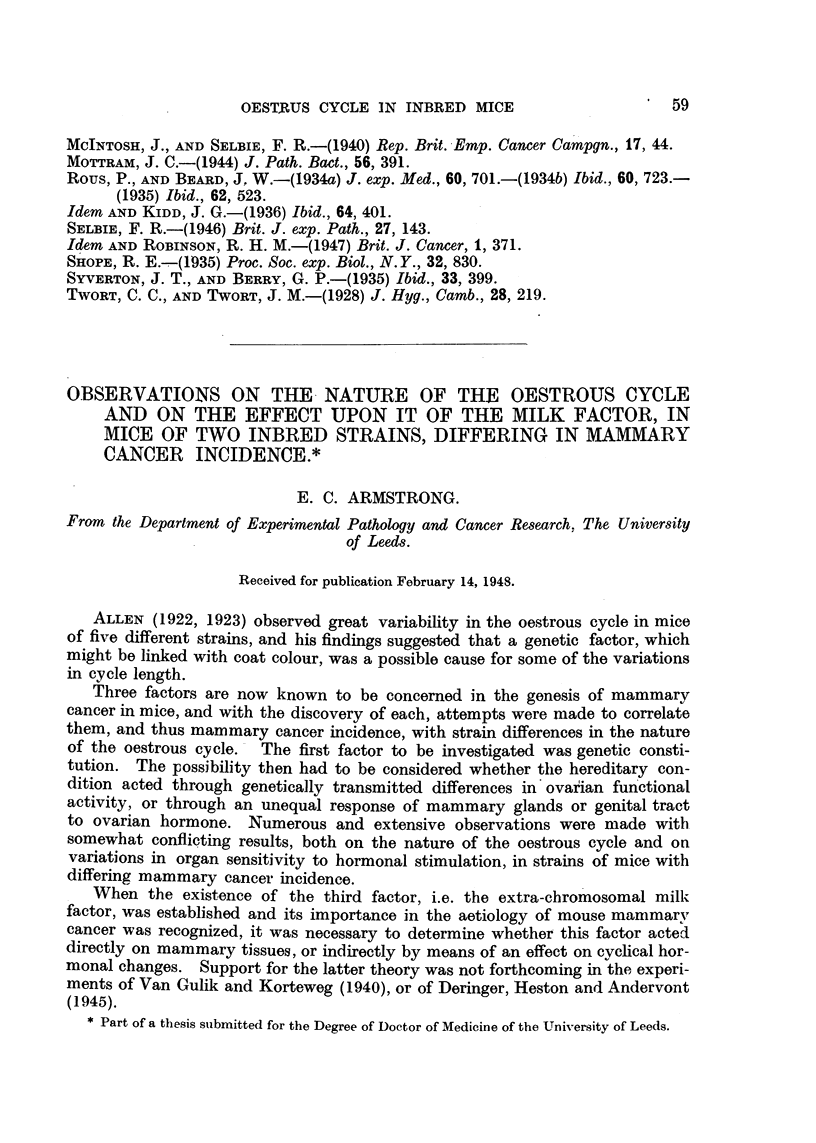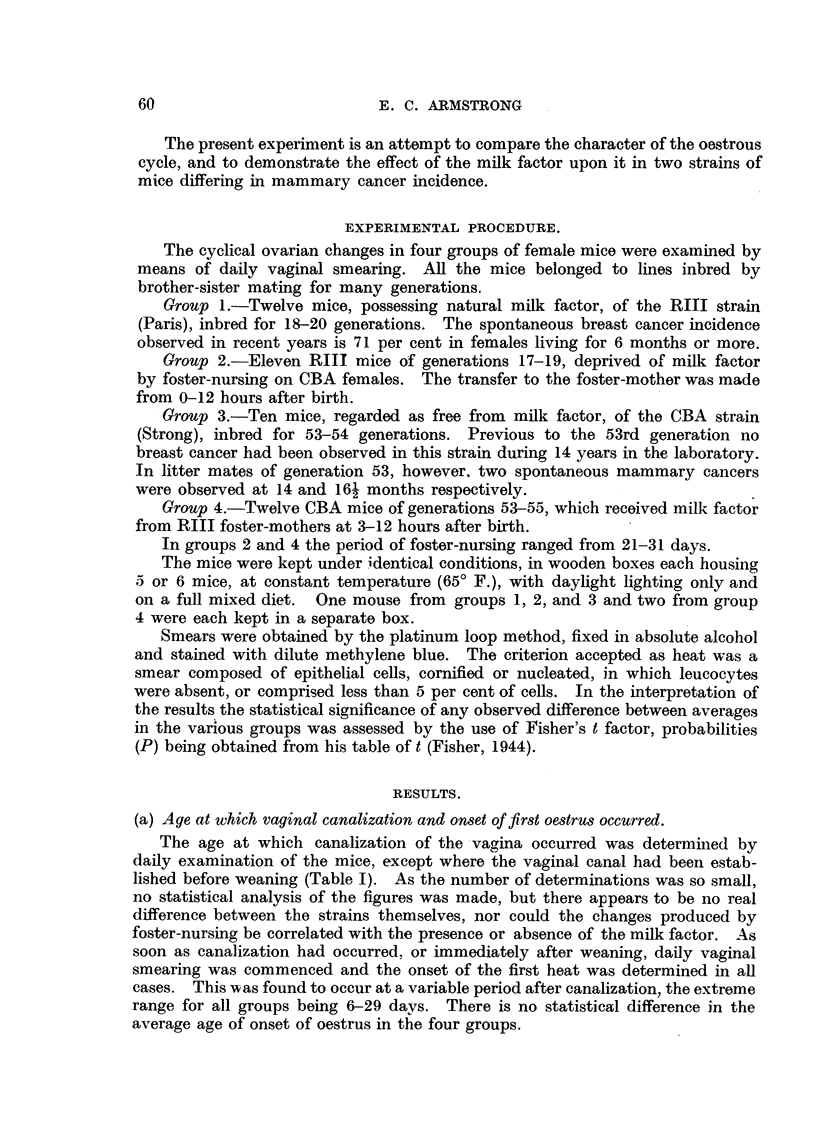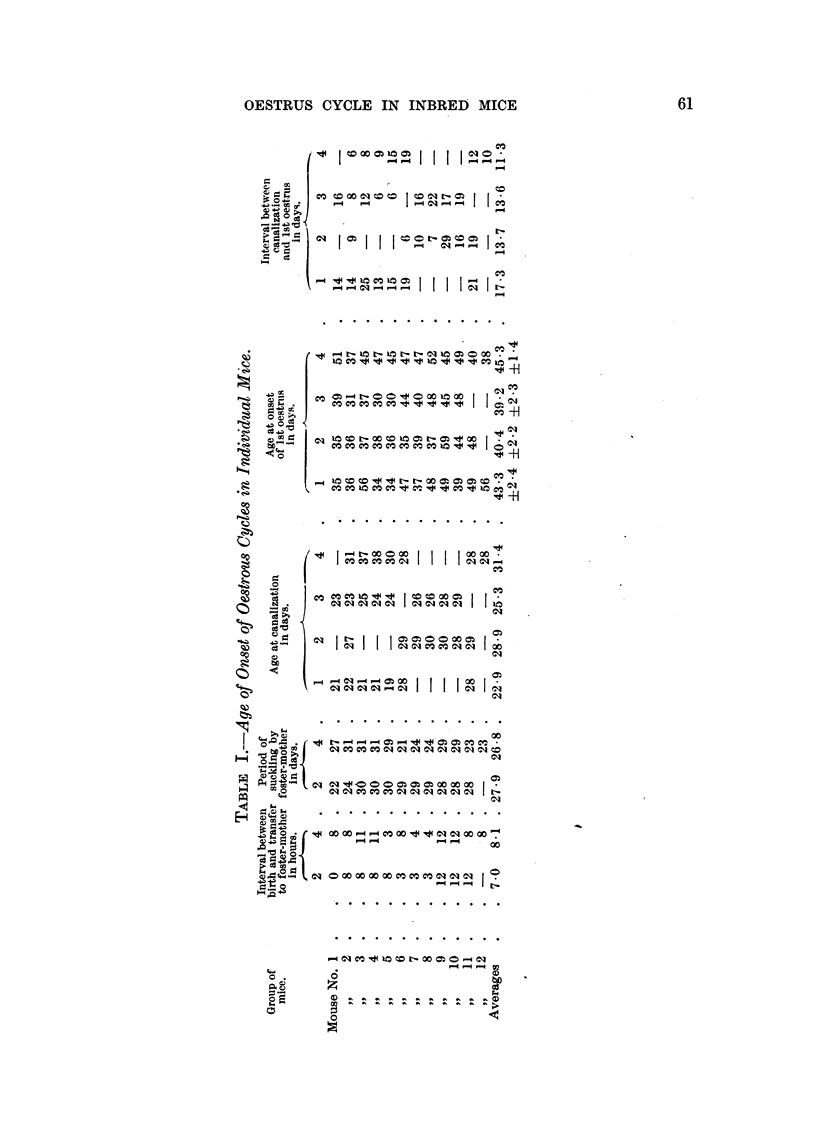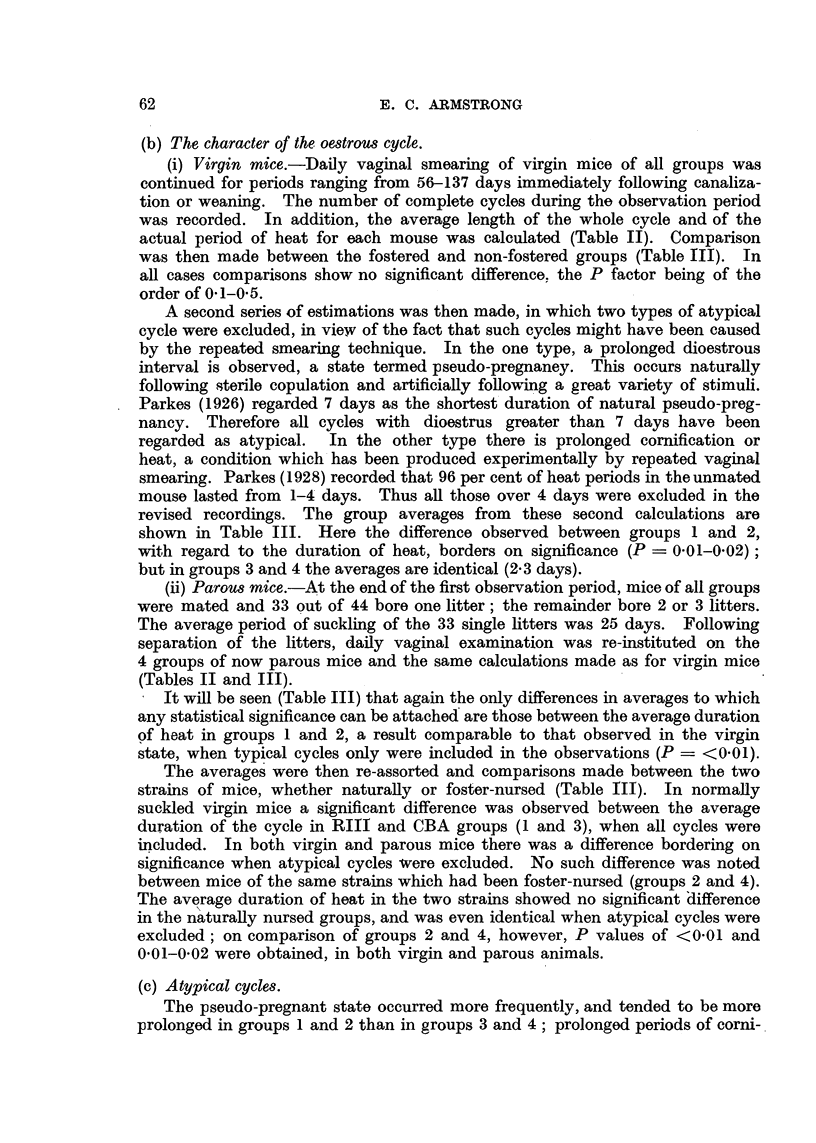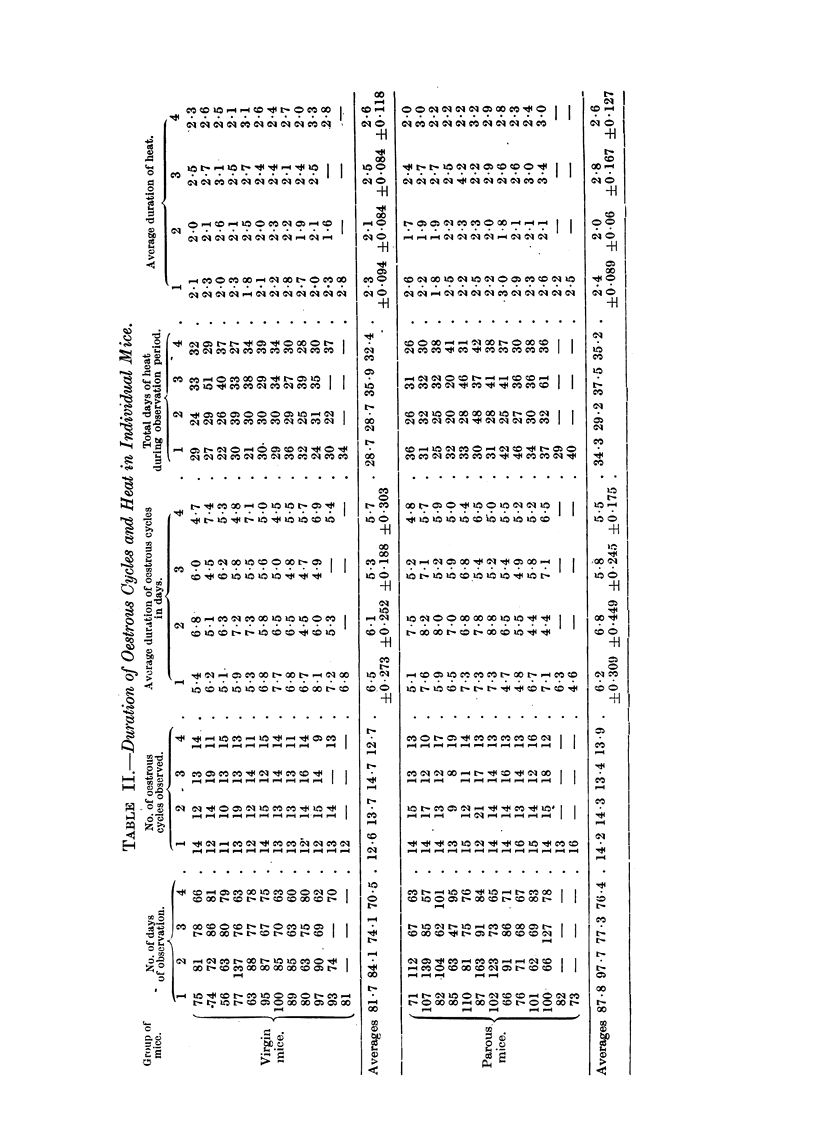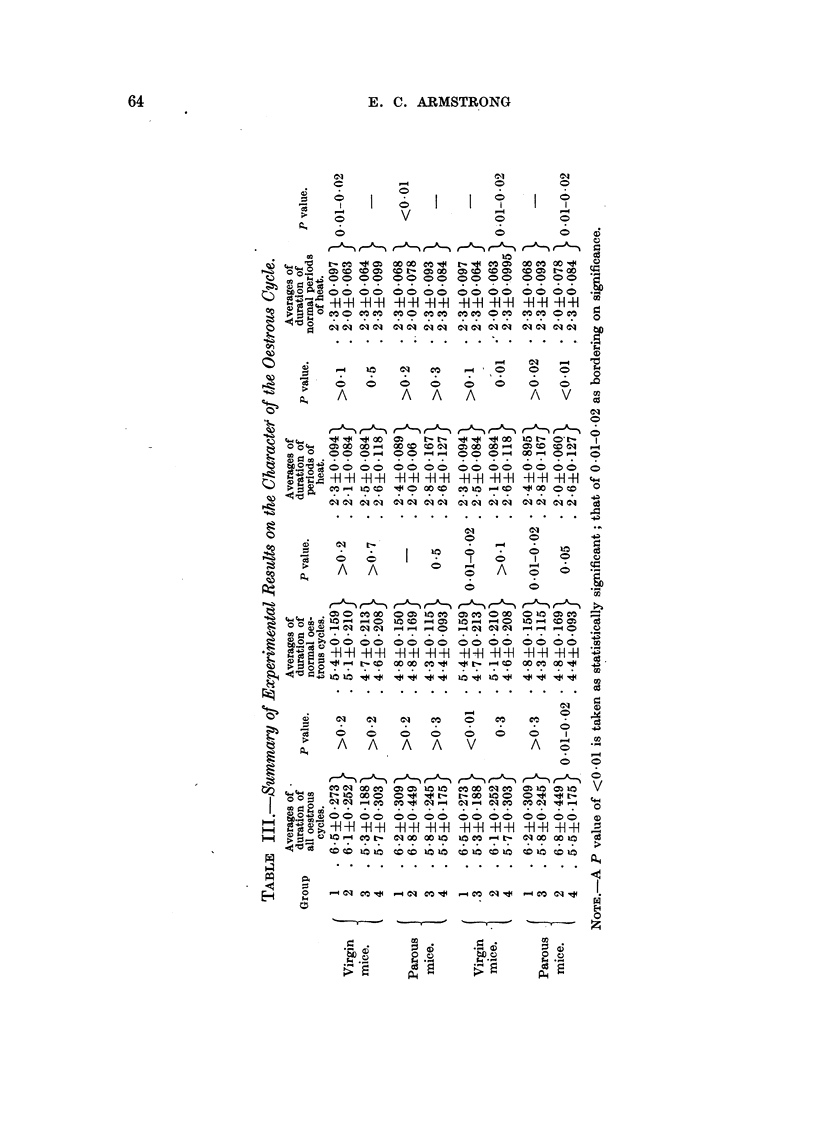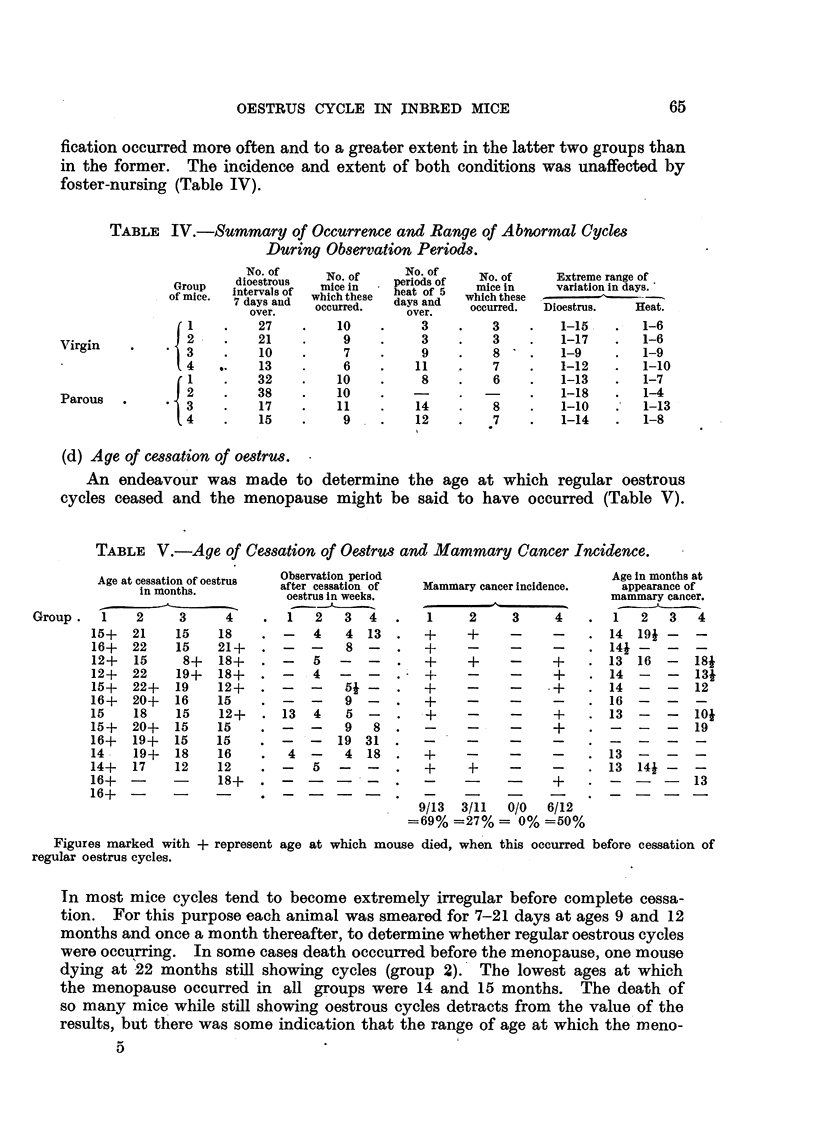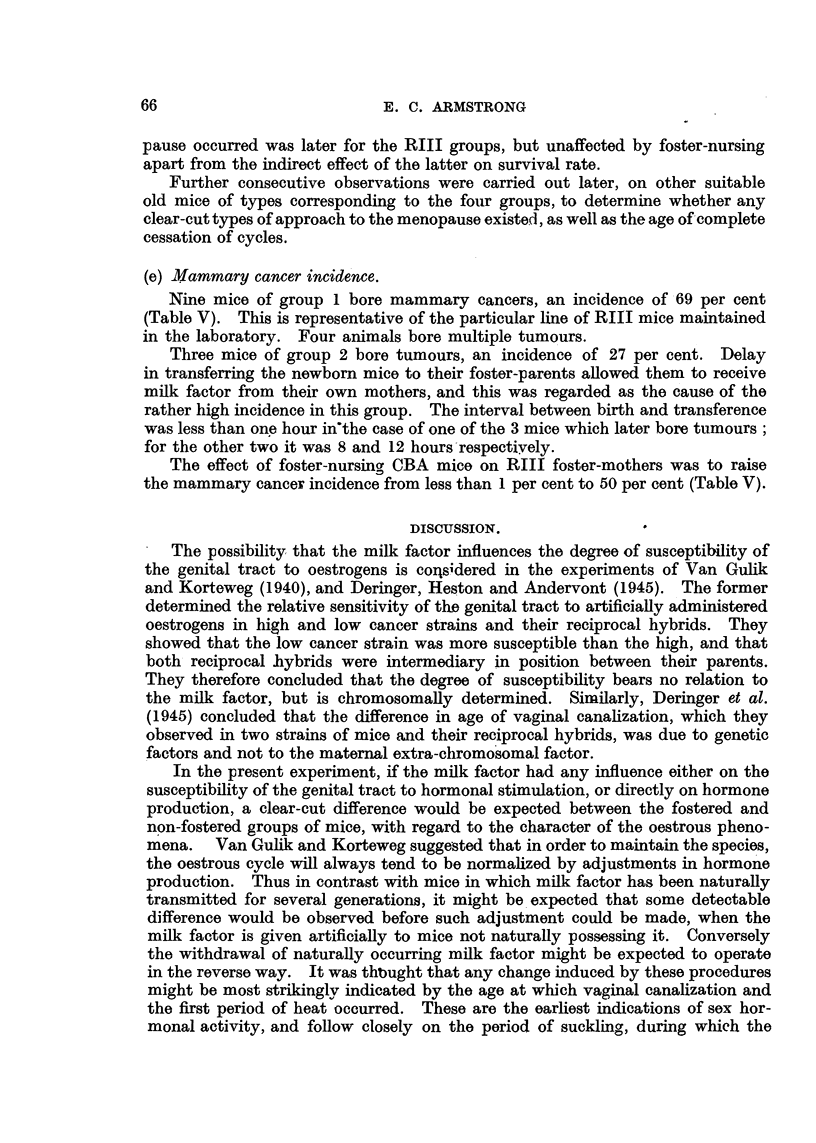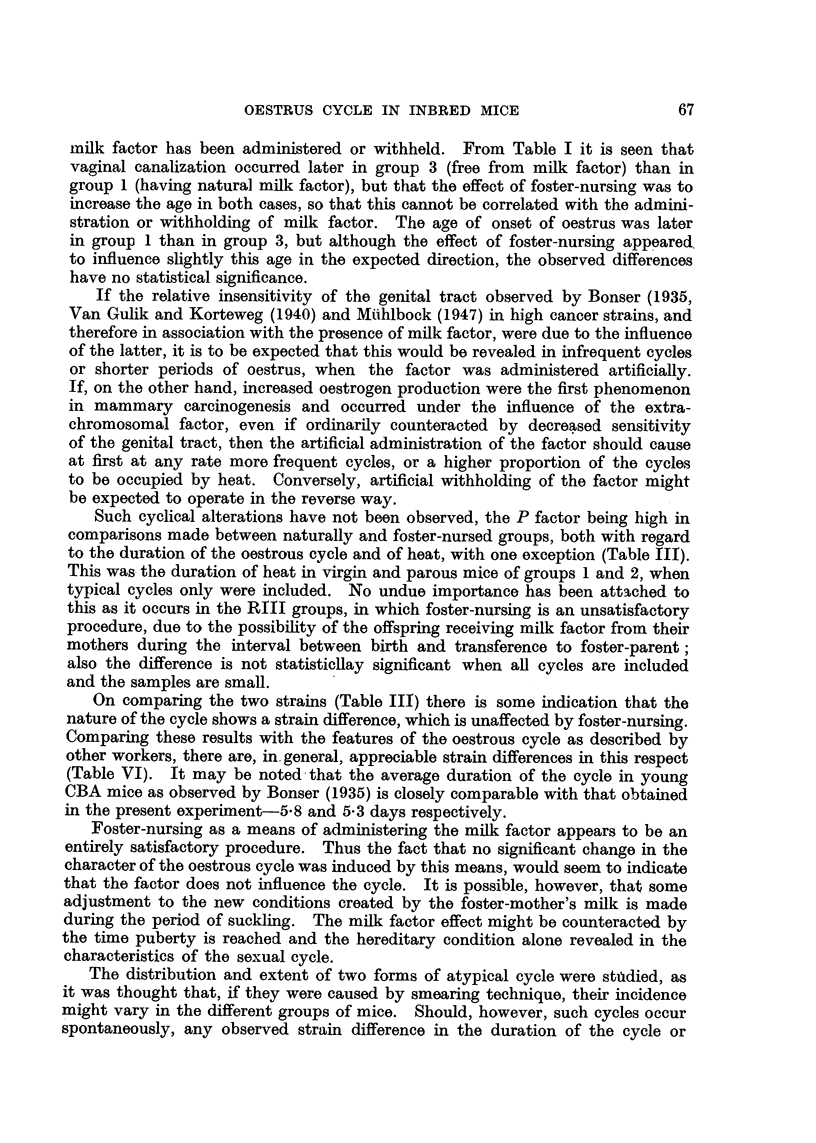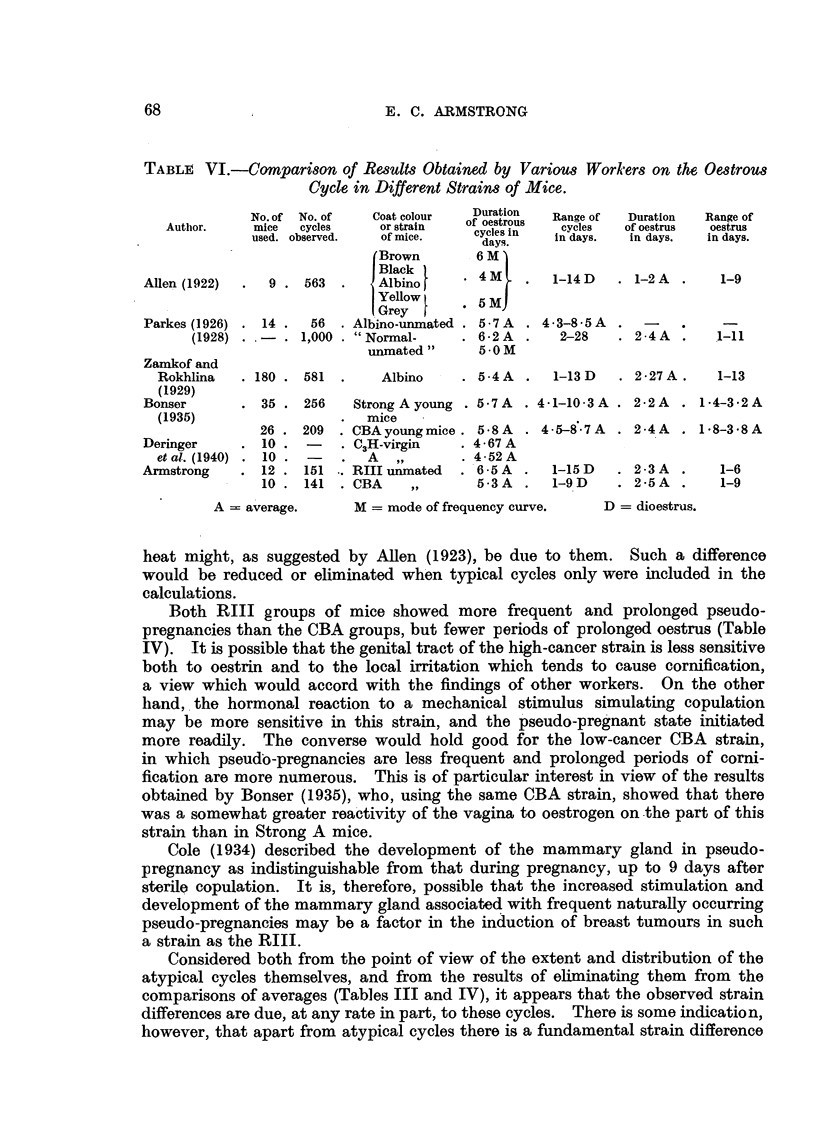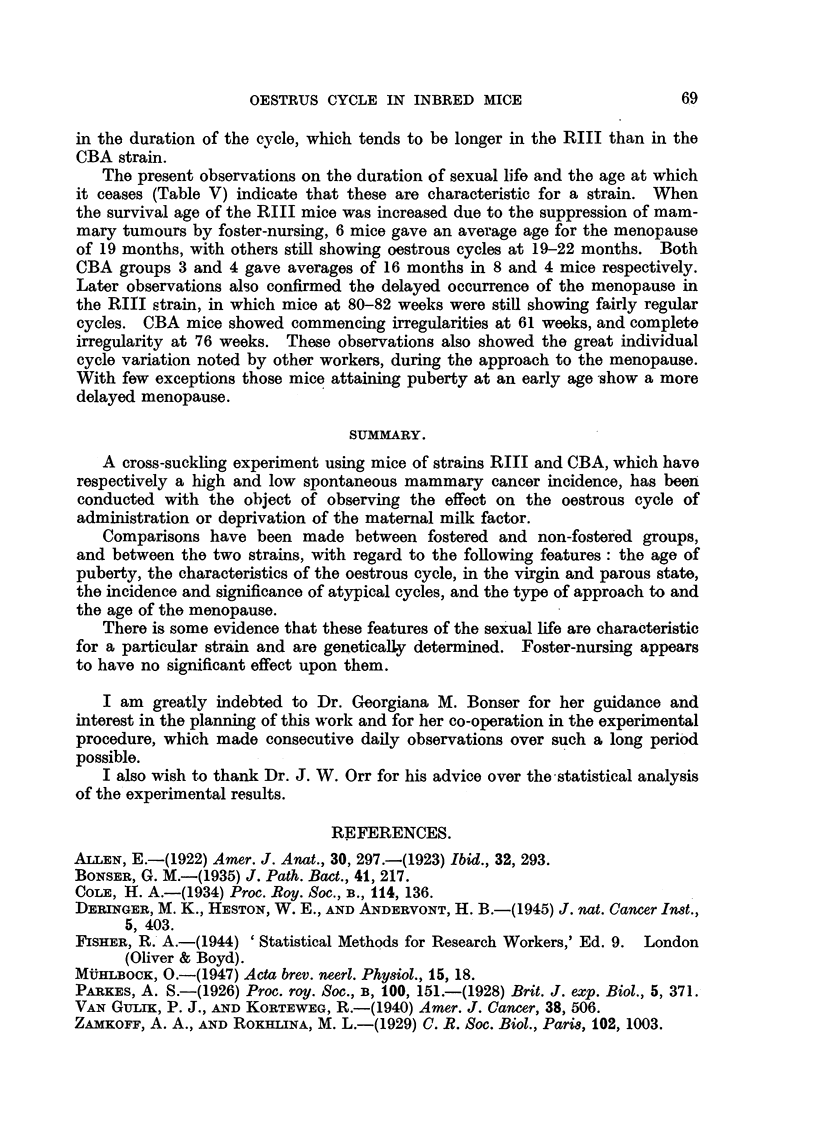# Observation on the Nature of the Oestrous Cycle and on the Effect Upon it of the Milk Factor, in Mice of Two Inbred Strains, Differing in Mammary cancer Incidence*

**DOI:** 10.1038/bjc.1948.8

**Published:** 1948-03

**Authors:** E. C. Armstrong


### OBSERVATIONS ON THE NATURE OF THE OESTROUS CYCLE AND ON THE EFFECT UPON IT OF THE MILK FACTOR, IN MICE OF TWO INBRED STRAINS, DIFFERING IN MAMMARY CANCER INCIDENCE.*

E. C. ARMSTRONG.

*From the Department of Experimental Pathology and Cancer Research, The University of Leeds.*

Received for publication February 14, 1948.

Allen (1922, 1923) observed great variability in the oestrous cycle in mice of five different strains, and his findings suggested that a genetic factor, which might be linked with coat colour, was a possible cause for some of the variations in cycle length.

Three factors are now known to be concerned in the genesis of mammary cancer in mice, and with the discovery of each, attempts were made to correlate them, and thus mammary cancer incidence, with strain differences in the nature of the oestrous cycle. The first factor to be investigated was genetic constitution. The possibility then had to be considered whether the hereditary condition acted through genetically transmitted differences in ovarian functional activity, or through an unequal response of mammary glands or genital tract to ovarian hormone. Numerous and extensive observations were made with somewhat conflicting results, both on the nature of the oestrous cycle and on variations in organ sensitivity to hormonal stimulation, in strains of mice with differing mammary cancer incidence.

When the existence of the third factor, i.e. the extra-chromosomal milk factor, was established and its importance in the aetiology of mouse mammary cancer was recognized, it was necessary to determine whether this factor acted directly on mammary tissues, or indirectly by means of an effect on cyclical hormonal changes. Support for the latter theory was not forthcoming in the experiments of Van Gulik and Korteweg (1940), or of Deringer, Heston and Andervont (1945).

* Part of a thesis submitted for the Degree of Doctor of Medicine of the University of Leeds.

The present experiment is an attempt to compare the character of the oestrous cycle, and to demonstrate the effect of the milk factor upon it in two strains of mice differing in mammary cancer incidence.

### EXPERIMENTAL PROCEDURE.

The cyclical ovarian changes in four groups of female mice were examined by means of daily vaginal smearing. All the mice belonged to lines inbred by brother-sister mating for many generations.

*Group* 1.—Twelve mice, possessing natural milk factor, of the RIII strain (Paris), inbred for 18–20 generations. The spontaneous breast cancer incidence observed in recent years is 71 per cent in females living for 6 months or more.

*Group* 2.—Eleven RIII mice of generations 17–19, deprived of milk factor by foster-nursing on CBA females. The transfer to the foster-mother was made from 0–12 hours after birth.

*Group* 3.—Ten mice, regarded as free from milk factor, of the CBA strain (Strong), inbred for 53–54 generations. Previous to the 53rd generation no breast cancer had been observed in this strain during 14 years in the laboratory. In litter mates of generation 53, however, two spontaneous mammary cancers were observed at 14 and 16½ months respectively.

*Group* 4.—Twelve CBA mice of generations 53–55, which received milk factor from RIII foster-mothers at 3–12 hours after birth.

In groups 2 and 4 the period of foster-nursing ranged from 21–31 days.

The mice were kept under identical conditions, in wooden boxes each housing 5 or 6 mice, at constant temperature (65° F.), with daylight lighting only and on a full mixed diet. One mouse from groups 1, 2, and 3 and two from group 4 were each kept in a separate box.

Smears were obtained by the platinum loop method, fixed in absolute alcohol and stained with dilute methylene blue. The criterion accepted as heat was a smear composed of epithelial cells, cornified or nucleated, in which leucocytes were absent, or comprised less than 5 per cent of cells. In the interpretation of the results the statistical significance of any observed difference between averages in the various groups was assessed by the use of Fisher's $t$ factor, probabilities ($P$) being obtained from his table of $t$ (Fisher, 1944).

### RESULTS.

(a) *Age at which vaginal canalization and onset of first oestrus occurred.*

The age at which canalization of the vagina occurred was determined by daily examination of the mice, except where the vaginal canal had been established before weaning (Table I). As the number of determinations was so small, no statistical analysis of the figures was made, but there appears to be no real difference between the strains themselves, nor could the changes produced by foster-nursing be correlated with the presence or absence of the milk factor. As soon as canalization had occurred, or immediately after weaning, daily vaginal smearing was commenced and the onset of the first heat was determined in all cases. This was found to occur at a variable period after canalization, the extreme range for all groups being 6–29 days. There is no statistical difference in the average age of onset of oestrus in the four groups.

(b) *The character of the oestrous cycle.*

(i) *Virgin mice.*—Daily vaginal smearing of virgin mice of all groups was continued for periods ranging from 56–137 days immediately following canalization or weaning. The number of complete cycles during the observation period was recorded. In addition, the average length of the whole cycle and of the actual period of heat for each mouse was calculated (Table II). Comparison was then made between the fostered and non-fostered groups (Table III). In all cases comparisons show no significant difference, the $P$ factor being of the order of 0·1–0·5.

A second series of estimations was then made, in which two types of atypical cycle were excluded, in view of the fact that such cycles might have been caused by the repeated smearing technique. In the one type, a prolonged dioestrous interval is observed, a state termed pseudo-pregnaney. This occurs naturally following sterile copulation and artificially following a great variety of stimuli. Parkes (1926) regarded 7 days as the shortest duration of natural pseudo-pregnancy. Therefore all cycles with dioestrus greater than 7 days have been regarded as atypical. In the other type there is prolonged cornification or heat, a condition which has been produced experimentally by repeated vaginal smearing. Parkes (1928) recorded that 96 per cent of heat periods in the unmated mouse lasted from 1–4 days. Thus all those over 4 days were excluded in the revised recordings. The group averages from these second calculations are shown in Table III. Here the difference observed between groups 1 and 2, with regard to the duration of heat, borders on significance ($P$ = 0·01–0·02); but in groups 3 and 4 the averages are identical (2·3 days).

(ii) *Parous mice.*—At the end of the first observation period, mice of all groups were mated and 33 out of 44 bore one litter; the remainder bore 2 or 3 litters. The average period of suckling of the 33 single litters was 25 days. Following separation of the litters, daily vaginal examination was re-instituted on the 4 groups of now parous mice and the same calculations made as for virgin mice (Tables II and III).

It will be seen (Table III) that again the only differences in averages to which any statistical significance can be attached are those between the average duration of heat in groups 1 and 2, a result comparable to that observed in the virgin state, when typical cycles only were included in the observations ($P = <0{\cdot}01$).

The averages were then re-assorted and comparisons made between the two strains of mice, whether naturally or foster-nursed (Table III). In normally suckled virgin mice a significant difference was observed between the average duration of the cycle in RIII and CBA groups (1 and 3), when all cycles were included. In both virgin and parous mice there was a difference bordering on significance when atypical cycles were excluded. No such difference was noted between mice of the same strains which had been foster-nursed (groups 2 and 4). The average duration of heat in the two strains showed no significant difference in the naturally nursed groups, and was even identical when atypical cycles were excluded; on comparison of groups 2 and 4, however, $P$ values of $<0{\cdot}01$ and 0·01–0·02 were obtained, in both virgin and parous animals.

(c) *Atypical cycles.*

The pseudo-pregnant state occurred more frequently, and tended to be more prolonged in groups 1 and 2 than in groups 3 and 4; prolonged periods of cornification occurred more often and to a greater extent in the latter two groups than in the former. The incidence and extent of both conditions was unaffected by foster-nursing (Table IV).

TABLE IV.—*Summary of Occurrence and Range of Abnormal Cycles During Observation Periods.*

| | Group of mice. | No. of dioestrous intervals of 7 days and over. | No. of mice in which these occurred. | No. of periods of heat of 5 days and over. | No. of mice in which these occurred. | Extreme range of variation in days. Dioestrus. | Heat. |
|---|---|---|---|---|---|---|---|
| Virgin | 1 | 27 | 10 | 3 | 3 | 1–15 | 1–6 |
| | 2 | 21 | 9 | 3 | 3 | 1–17 | 1–6 |
| | 3 | 10 | 7 | 9 | 8 | 1–9 | 1–9 |
| | 4 | 13 | 6 | 11 | 7 | 1–12 | 1–10 |
| Parous | 1 | 32 | 10 | 8 | 6 | 1–13 | 1–7 |
| | 2 | 38 | 10 | — | — | 1–18 | 1–4 |
| | 3 | 17 | 11 | 14 | 8 | 1–10 | 1–13 |
| | 4 | 15 | 9 | 12 | 7 | 1–14 | 1–8 |

(d) *Age of cessation of oestrus.*

An endeavour was made to determine the age at which regular oestrous cycles ceased and the menopause might be said to have occurred (Table V).

TABLE V.—*Age of Cessation of Oestrus and Mammary Cancer Incidence.*

| | Age at cessation of oestrus in months. | | | | Observation period after cessation of oestrus in weeks. | | | | Mammary cancer incidence. | | | | Age in months at appearance of mammary cancer. | | | |
|---|---|---|---|---|---|---|---|---|---|---|---|---|---|---|---|---|
| Group | 1 | 2 | 3 | 4 | 1 | 2 | 3 | 4 | 1 | 2 | 3 | 4 | 1 | 2 | 3 | 4 |
| | 15+ | 21 | 15 | 18 | — | 4 | 4 | 13 | + | + | — | — | 14 | 19½ | — | — |
| | 16+ | 22 | 15 | 21+ | — | — | 8 | — | + | — | — | — | 14½ | — | — | — |
| | 12+ | 15 | 8+ | 18+ | — | 5 | — | — | + | + | — | + | 13 | 16 | — | 18½ |
| | 12+ | 22 | 19+ | 18+ | — | 4 | — | — | + | — | — | + | 14 | — | — | 13½ |
| | 15+ | 22+ | 19 | 12+ | — | — | 5½ | — | + | — | — | + | 14 | — | — | 12 |
| | 16+ | 20+ | 16 | 15 | — | — | 9 | — | + | — | — | — | 16 | — | — | — |
| | 15 | 18 | 15 | 12+ | 13 | 4 | 5 | — | + | — | — | + | 13 | — | — | 10½ |
| | 15+ | 20+ | 15 | 15 | — | — | 9 | 8 | — | — | — | + | — | — | — | 19 |
| | 16+ | 19+ | 15 | 15 | — | — | 19 | 31 | — | — | — | — | — | — | — | — |
| | 14 | 19+ | 18 | 16 | 4 | — | 4 | 18 | + | — | — | — | 13 | — | — | — |
| | 14+ | 17 | 12 | 12 | — | 5 | — | — | + | + | — | — | 13 | 14½ | — | — |
| | 16+ | — | — | 18+ | — | — | — | — | — | — | — | + | — | — | — | 13 |
| | 16+ | — | — | — | — | — | — | — | — | — | — | — | — | — | — | — |
| | | | | | | | | | 9/13 =69% | 3/11 =27% | 0/0 = 0% | 6/12 =50% | | | | |

Figures marked with + represent age at which mouse died, when this occurred before cessation of regular oestrus cycles.

In most mice cycles tend to become extremely irregular before complete cessation. For this purpose each animal was smeared for 7–21 days at ages 9 and 12 months and once a month thereafter, to determine whether regular oestrous cycles were occurring. In some cases death occcurred before the menopause, one mouse dying at 22 months still showing cycles (group 2). The lowest ages at which the menopause occurred in all groups were 14 and 15 months. The death of so many mice while still showing oestrous cycles detracts from the value of the results, but there was some indication that the range of age at which the menopause occurred was later for the RIII groups, but unaffected by foster-nursing apart from the indirect effect of the latter on survival rate.

Further consecutive observations were carried out later, on other suitable old mice of types corresponding to the four groups, to determine whether any clear-cut types of approach to the menopause existed, as well as the age of complete cessation of cycles.

(e) *Mammary cancer incidence.*

Nine mice of group 1 bore mammary cancers, an incidence of 69 per cent (Table V). This is representative of the particular line of RIII mice maintained in the laboratory. Four animals bore multiple tumours.

Three mice of group 2 bore tumours, an incidence of 27 per cent. Delay in transferring the newborn mice to their foster-parents allowed them to receive milk factor from their own mothers, and this was regarded as the cause of the rather high incidence in this group. The interval between birth and transference was less than one hour in the case of one of the 3 mice which later bore tumours; for the other two it was 8 and 12 hours respectively.

The effect of foster-nursing CBA mice on RIII foster-mothers was to raise the mammary cancer incidence from less than 1 per cent to 50 per cent (Table V).

### DISCUSSION.

The possibility that the milk factor influences the degree of susceptibility of the genital tract to oestrogens is considered in the experiments of Van Gulik and Korteweg (1940), and Deringer, Heston and Andervont (1945). The former determined the relative sensitivity of the genital tract to artificially administered oestrogens in high and low cancer strains and their reciprocal hybrids. They showed that the low cancer strain was more susceptible than the high, and that both reciprocal hybrids were intermediary in position between their parents. They therefore concluded that the degree of susceptibility bears no relation to the milk factor, but is chromosomally determined. Similarly, Deringer *et al.* (1945) concluded that the difference in age of vaginal canalization, which they observed in two strains of mice and their reciprocal hybrids, was due to genetic factors and not to the maternal extra-chromosomal factor.

In the present experiment, if the milk factor had any influence either on the susceptibility of the genital tract to hormonal stimulation, or directly on hormone production, a clear-cut difference would be expected between the fostered and non-fostered groups of mice, with regard to the character of the oestrous phenomena. Van Gulik and Korteweg suggested that in order to maintain the species, the oestrous cycle will always tend to be normalized by adjustments in hormone production. Thus in contrast with mice in which milk factor has been naturally transmitted for several generations, it might be expected that some detectable difference would be observed before such adjustment could be made, when the milk factor is given artificially to mice not naturally possessing it. Conversely the withdrawal of naturally occurring milk factor might be expected to operate in the reverse way. It was thought that any change induced by these procedures might be most strikingly indicated by the age at which vaginal canalization and the first period of heat occurred. These are the earliest indications of sex hormonal activity, and follow closely on the period of suckling, during which the milk factor has been administered or withheld. From Table I it is seen that vaginal canalization occurred later in group 3 (free from milk factor) than in group 1 (having natural milk factor), but that the effect of foster-nursing was to increase the age in both cases, so that this cannot be correlated with the administration or withholding of milk factor. The age of onset of oestrus was later in group 1 than in group 3, but although the effect of foster-nursing appeared to influence slightly this age in the expected direction, the observed differences have no statistical significance.

If the relative insensitivity of the genital tract observed by Bonser (1935, Van Gulik and Korteweg (1940) and Mühlbock (1947) in high cancer strains, and therefore in association with the presence of milk factor, were due to the influence of the latter, it is to be expected that this would be revealed in infrequent cycles or shorter periods of oestrus, when the factor was administered artificially. If, on the other hand, increased oestrogen production were the first phenomenon in mammary carcinogenesis and occurred under the influence of the extrachromosomal factor, even if ordinarily counteracted by decreased sensitivity of the genital tract, then the artificial administration of the factor should cause at first at any rate more frequent cycles, or a higher proportion of the cycles to be occupied by heat. Conversely, artificial withholding of the factor might be expected to operate in the reverse way.

Such cyclical alterations have not been observed, the *P* factor being high in comparisons made between naturally and foster-nursed groups, both with regard to the duration of the oestrous cycle and of heat, with one exception (Table III). This was the duration of heat in virgin and parous mice of groups 1 and 2, when typical cycles only were included. No undue importance has been attached to this as it occurs in the RIII groups, in which foster-nursing is an unsatisfactory procedure, due to the possibility of the offspring receiving milk factor from their mothers during the interval between birth and transference to foster-parent; also the difference is not statisticllay significant when all cycles are included and the samples are small.

On comparing the two strains (Table III) there is some indication that the nature of the cycle shows a strain difference, which is unaffected by foster-nursing. Comparing these results with the features of the oestrous cycle as described by other workers, there are, in general, appreciable strain differences in this respect (Table VI). It may be noted that the average duration of the cycle in young CBA mice as observed by Bonser (1935) is closely comparable with that obtained in the present experiment—5·8 and 5·3 days respectively.

Foster-nursing as a means of administering the milk factor appears to be an entirely satisfactory procedure. Thus the fact that no significant change in the character of the oestrous cycle was induced by this means, would seem to indicate that the factor does not influence the cycle. It is possible, however, that some adjustment to the new conditions created by the foster-mother's milk is made during the period of suckling. The milk factor effect might be counteracted by the time puberty is reached and the hereditary condition alone revealed in the characteristics of the sexual cycle.

The distribution and extent of two forms of atypical cycle were studied, as it was thought that, if they were caused by smearing technique, their incidence might vary in the different groups of mice. Should, however, such cycles occur spontaneously, any observed strain difference in the duration of the cycle or

TABLE VI.—*Comparison of Results Obtained by Various Workers on the Oestrous Cycle in Different Strains of Mice.*

| Author. | No. of mice used. | No. of cycles observed. | Coat colour or strain of mice. | Duration of oestrous cycles in days. | Range of cycles in days. | Duration of oestrus in days. | Range of oestrus in days. |
|---|---|---|---|---|---|---|---|
| Allen (1922) | 9 | 563 | Brown | 6 M | 1–14 D | 1–2 A | 1–9 |
| | | | Black, Albino | 4 M | | | |
| | | | Yellow, Grey | 5 M | | | |
| Parkes (1926) | 14 | 56 | Albino-unmated | 5·7 A | 4·3–8·5 A | — | — |
| (1928) | — | 1,000 | "Normal-unmated" | 6·2 A<br>5·0 M | 2–28 | 2·4 A | 1–11 |
| Zamkof and Rokhlina (1929) | 180 | 581 | Albino | 5·4 A | 1–13 D | 2·27 A | 1–13 |
| Bonser (1935) | 35 | 256 | Strong A young mice | 5·7 A | 4·1–10·3 A | 2·2 A | 1·4–3·2 A |
| | 26 | 209 | CBA young mice | 5·8 A | 4·5–8·7 A | 2·4 A | 1·8–3·8 A |
| Deringer *et al.* (1940) | 10 | — | $C_3H$-virgin | 4·67 A | | | |
| | 10 | — | A ,, | 4·52 A | | | |
| Armstrong | 12 | 151 | RIII unmated | 6·5 A | 1–15 D | 2·3 A | 1–6 |
| | 10 | 141 | CBA ,, | 5·3 A | 1–9 D | 2·5 A | 1–9 |

A = average. M = mode of frequency curve. D = dioestrus.

heat might, as suggested by Allen (1923), be due to them. Such a difference would be reduced or eliminated when typical cycles only were included in the calculations.

Both RIII groups of mice showed more frequent and prolonged pseudo-pregnancies than the CBA groups, but fewer periods of prolonged oestrus (Table IV). It is possible that the genital tract of the high-cancer strain is less sensitive both to oestrin and to the local irritation which tends to cause cornification, a view which would accord with the findings of other workers. On the other hand, the hormonal reaction to a mechanical stimulus simulating copulation may be more sensitive in this strain, and the pseudo-pregnant state initiated more readily. The converse would hold good for the low-cancer CBA strain, in which pseudo-pregnancies are less frequent and prolonged periods of cornification are more numerous. This is of particular interest in view of the results obtained by Bonser (1935), who, using the same CBA strain, showed that there was a somewhat greater reactivity of the vagina to oestrogen on the part of this strain than in Strong A mice.

Cole (1934) described the development of the mammary gland in pseudo-pregnancy as indistinguishable from that during pregnancy, up to 9 days after sterile copulation. It is, therefore, possible that the increased stimulation and development of the mammary gland associated with frequent naturally occurring pseudo-pregnancies may be a factor in the induction of breast tumours in such a strain as the RIII.

Considered both from the point of view of the extent and distribution of the atypical cycles themselves, and from the results of eliminating them from the comparisons of averages (Tables III and IV), it appears that the observed strain differences are due, at any rate in part, to these cycles. There is some indication, however, that apart from atypical cycles there is a fundamental strain difference in the duration of the cycle, which tends to be longer in the RIII than in the CBA strain.

The present observations on the duration of sexual life and the age at which it ceases (Table V) indicate that these are characteristic for a strain. When the survival age of the RIII mice was increased due to the suppression of mammary tumours by foster-nursing, 6 mice gave an average age for the menopause of 19 months, with others still showing oestrous cycles at 19–22 months. Both CBA groups 3 and 4 gave averages of 16 months in 8 and 4 mice respectively. Later observations also confirmed the delayed occurrence of the menopause in the RIII strain, in which mice at 80–82 weeks were still showing fairly regular cycles. CBA mice showed commencing irregularities at 61 weeks, and complete irregularity at 76 weeks. These observations also showed the great individual cycle variation noted by other workers, during the approach to the menopause. With few exceptions those mice attaining puberty at an early age show a more delayed menopause.

### SUMMARY.

A cross-suckling experiment using mice of strains RIII and CBA, which have respectively a high and low spontaneous mammary cancer incidence, has been conducted with the object of observing the effect on the oestrous cycle of administration or deprivation of the maternal milk factor.

Comparisons have been made between fostered and non-fostered groups, and between the two strains, with regard to the following features : the age of puberty, the characteristics of the oestrous cycle, in the virgin and parous state, the incidence and significance of atypical cycles, and the type of approach to and the age of the menopause.

There is some evidence that these features of the sexual life are characteristic for a particular strain and are genetically determined. Foster-nursing appears to have no significant effect upon them.

I am greatly indebted to Dr. Georgiana M. Bonser for her guidance and interest in the planning of this work and for her co-operation in the experimental procedure, which made consecutive daily observations over such a long period possible.

I also wish to thank Dr. J. W. Orr for his advice over the statistical analysis of the experimental results.

### REFERENCES.

Allen, E.—(1922) *Amer. J. Anat.*, **30**, 297.—(1923) *Ibid.*, **32**, 293.
Bonser, G. M.—(1935) *J. Path. Bact.*, **41**, 217.
Cole, H. A.—(1934) *Proc. Roy. Soc.*, B., **114**, 136.
Deringer, M. K., Heston, W. E., and Andervont, H. B.—(1945) *J. nat. Cancer Inst.*, **5**, 403.
Fisher, R. A.—(1944) 'Statistical Methods for Research Workers,' Ed. 9. London (Oliver & Boyd).
Mühlbock, O.—(1947) *Acta brev. neerl. Physiol.*, **15**, 18.
Parkes, A. S.—(1926) *Proc. roy. Soc.*, B, **100**, 151.—(1928) *Brit. J. exp. Biol.*, **5**, 371.
Van Gulik, P. J., and Korteweg, R.—(1940) *Amer. J. Cancer*, **38**, 506.
Zamkoff, A. A., and Rokhlina, M. L.—(1929) *C. R. Soc. Biol., Paris*, **102**, 1003.